# Are Total and Individual Dietary Lignans Related to Cardiovascular Disease and Its Risk Factors in Postmenopausal Women? A Nationwide Study

**DOI:** 10.3390/nu10070865

**Published:** 2018-07-04

**Authors:** Anna Maria Witkowska, Anna Waśkiewicz, Małgorzata Elżbieta Zujko, Danuta Szcześniewska, Urszula Stepaniak, Andrzej Pająk, Wojciech Drygas

**Affiliations:** 1 Department of Food Biotechnology, Faculty of Health Sciences, Medical University of Bialystok, Szpitalna 37, 15-295 Bialystok, Poland; malgorzata.zujko@umb.edu.pl; 2 Department of Epidemiology, Cardiovascular Disease Prevention and Health Promotion, Institute of Cardiology, Alpejska 42, 04-628 Warsaw, Poland; awaskiewicz@ikard.pl (A.W.); dszczesniewska@ikard.pl (D.S.); wdrygas@ikard.pl (W.D.); 3 Department of Epidemiology and Population Studies, Faculty of Health Sciences, Institute of Public Health, Jagiellonian University Medical College, Grzegórzecka 20, 31-531 Krakow, Poland; urszula.stepaniak@uj.edu.pl (U.S.); andrzej.pajak@uj.edu.pl (A.P.); 4 Department of Social and Preventive Medicine, Faculty of Health Sciences, Medical University of Lodz, Hallera 1, 90-001 Lodz, Poland

**Keywords:** lignan, lariciresinol, postmenopausal, cardiovascular disease, hypercholesterolemia, hypertension, central obesity

## Abstract

The study objectives were to examine total and individual lignan intakes and their dietary sources in postmenopausal Polish women and to investigate the relationship between lignan intake and the prevalence of cardiovascular disease (CVD), hypertension, hypercholesterolemia and central obesity. A total of 2599 postmenopausal women, participants of the Multi-centre National Population Health Examination Surveys (WOBASZ and WOBASZ II) were selected. Of them, 916 had a history of CVD. Nutritional data were collected using a single 24-h dietary recall. Data on lignan content in food, i.e., lariciresinol (LARI), matairesinol (MAT), pinoresinol (PINO) and secoisolariciresinol (SECO), were collected from the available lignan databases. In postmenopausal women, total and individual lignan intakes (SECO, PINO, MAT) were not associated with the prevalence of CVD and its risk factors. The intake of LARI was linked by 30% to the reduced odds for hypercholestrolemia. This study reinforces the existing concept that dietary total lignans are not associated with the prevalence of CVD, and provides further evidence that they are not linked to CVD risk factors such as hypertension, hypercholesterolemia and central obesity. However, the intake of LARI should be taken into consideration in further studies with regard to its potentially beneficial effect in hypercholesterolemia.

## 1. Introduction

Lignans are plant-derived diphenolic compounds formed by the dimerization of two cinnamic acid residues that are widely distributed in seeds, grains, legumes, fruit and vegetables. The most common food representatives of lignans are secoisolariciresinol, matairesinol, pinoresinol and lariciresinol. Compared to flavonoids and phenolic acids, which are present in food in larger amounts [1], the usual intake of lignans is minor, ranging between ~1–1.6 mg/day when these four lignans are taken into account [2]. Dietary sources of total and individual lignans may vary among countries and are determined by the habitual patterns of food consumption.

In the human gastrointestinal tract, the enterolignan precursors, i.e., secoisolariciresinol, matairesinol, pinoresinol, lariciresinol, are metabolized by anaerobic bacteria to enterolignans, which demonstrate antioxidant properties as well as estrogenic activity [3,4]. In addition, as it has been recently reviewed, lignans possess anti-aging properties [5]. These characteristics of lignans may be important for the prevention of cardiovascular disease (CVD) in postmenopausal women, who manifest higher prevalence of CVD in contrast to premenopausal females [6]. In the intervention studies, lignan supplements demonstrated lowering effects on plasma lipids [7]. Epidemiological evidence, however, is inconclusive. Some studies suggest potential advantages of habitual intakes of total or individual lignans [8,9,10]. An inverse association between the high lignan intake and hypertension [8] and the incidence of CVD [9] was found. The intake of an individual lignan, matairesinol, was inversely associated with the mortality due to CVD [10]. Others do not support the concept of reduced mortality due to dietary phytoestrogen intake [11]. Only slight associations of total lignan intakes with reduced blood pressure, lower prevalence of hypertension, lower triglyceride concentration and reduced aortic stiffness have been described [12]. In turn, individual lignans such as matairesinol and secoisolariciresinol have been favorably related to the parameters of vascular inflammation and endothelial dysfunction [13]. These ambiguous findings of the previous studies warrant further research regarding dietary lignan intakes and their presumable beneficial role in CVD.

The current study was designed to assess total and individual lignan intakes and their dietary sources in postmenopausal Polish women, and to assess the relationship between lignan intake and the prevalence of hypercholesterolemia, hypertension, central obesity and cardiovascular disease.

## 2. Materials and Methods

### 2.1. Participants

**Figure 1:**
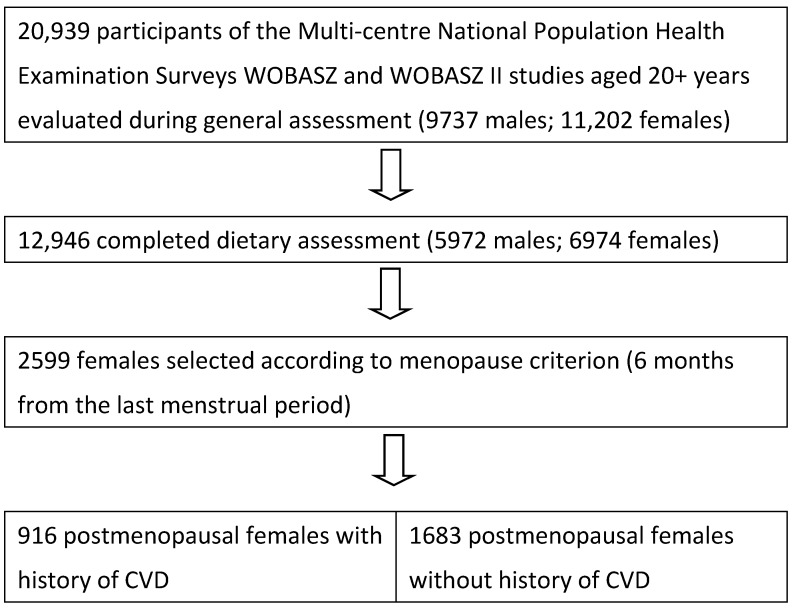
Flow-chart of study participants. CVD: cardiovascular disease.

The Multi-centre National Population Health Examination Surveys, WOBASZ (2003–2005) and WOBASZ II (2013–2014) were the two largest population-based cross-sectional studies carried out in Poland by the National Institute of Cardiology, Warsaw, Poland, in collaboration with five Polish medical universities [14,15,16]. These surveys evaluated representative random samples of the total Polish population in ~21,000 men and women aged 20–74 years (WOBASZ) and 20+ years. (WOBASZ II). All subjects gave their informed consent for inclusion before they participated in the studies. The studies were conducted in accordance with the Declaration of Helsinki, and the protocol was approved by the Ethics Committee of the Institute of Cardiology (WOBASZ, No. 708) and (WOBASZ II, No. 1344). Nutrition studies were conducted in the approximately 62% sample of the study population (Figure 1). From these participants, 2599 postmenopausal women were selected based on the question referring to the occurrence of natural menopause and completed records. The same menopause criterion of six months from the termination of menstruation was adopted in both studies. The exclusion criteria were pregnancy and surgical menopause. Then, a group of 916 women with a history of CVD was extracted from the general study group of postmenopausal women. The remaining 1683 women without past history of CVD served as controls. Data on hypertension, hypercholesterolemia and obesity were obtained for all 2599 women. The flow-chart of the study participants is provided in Figure 1.

### 2.2. Data Collection

Data on the level of education (under middle, middle, high), family history of CVD (yes/no), health status (hypertension, myocardial infarction, stroke, diabetes), menopause-hormone therapy (yes/no), leisure-time physical activity (low, middle, high), alcohol intake (pure ethanol g/day) and smoking habit (current smokers, past smokers, never smokers), and the type and dose of medication being taken were collected from the standardized questionnaire designed for the WOBASZ study. Cardiovascular disease classification has been adopted in accordance with the World Health Organization (WHO) [17], where CVDs due to atherosclerosis include ischaemic heart disease or coronary artery disease (e.g., myocardial infarction), cerebrovascular disease (e.g., stroke), diseases of the aorta and arteries, including hypertension and peripheral vascular disease.

Measurements of systolic blood pressure (SBP) and diastolic blood pressure (DBP) were taken three times on the right arm after 5 min of resting in a sitting position in one-minute intervals and the final SBP and DBP were calculated as an average of the second and third measurements. Hypertension was recognized for SBP ≥ 140 mmHg or DBP ≥ 90 mmHg or when antihypertensive drugs were used. Hypercholesterolemia was diagnosed if total blood cholesterol was ≥5 mmol/L or LDL-cholesterol levels were ≥3 mmol/L or the participant was taking a lipid-lowering medication. Diabetes was deemed present when blood glucose level was ≥7.0 mmol/L or diabetes was declared in an interview.

Body measurements, such as height, body mass and waist circumference, were taken by the personnel trained in standard procedures. The body mass index (BMI) was calculated from weight in kilograms divided by the square of height in meters. The participants were diagnosed with central obesity, when their waist circumference was ≥80 cm, which is a cut off value for European females. Biochemical analyses, i.e., fasting glucose and total cholesterol, were carried out at a single location, Diagnostyka Central Laboratory at the Institute of Cardiology in Warsaw. A general description of the study group stratified by CVD status has been given in Table 1. A more detailed description of the CVD women and those without past history of CVD has been published elsewhere [18].

### 2.3. Dietary Assessment

Nutritional data were collected by qualified interviewers using a single 24-h dietary recall, which revealed that 367 dishes, food items and beverages consumed by the participants were lignan sources. Individual components of complex dishes were extracted using dish recipes from the Polish Food Composition Tables [19]. These recipes give the amounts of food items required for 100 g dish portion, with consideration of yield factors. The quantitative composition of plant components of ready-to-eat foods was obtained from food labels. For fruit yoghurt, for example, a typical amount of 5% added fruit was included in the calculation. Data on tea type (black, green, herbal) were not collected. By default, it was assumed that black tea, the most popular tea in Poland, would be consumed.

### 2.4. Estimation of Total and Individual Lignan Intakes

Data on food content of lignans: lariciresinol (LARI), matairesinol (MAT), pinoresinol (PINO) and secoisolariciresinol (SECO) were collected from the available lignan databases [20,21,22]. The primary source of lignan values in this study was Dutch lignan database [20]. The lignan contents of beverages, nuts, seeds, and oils were taken from Kuhnle et al. [21]. The Thompson et al. [22] database was used when data on lignan content were missing. PINO content in onions, parsnips, fruit jam, apples, LARI content in parsnips and jam, and MAT content in onion, pepper, cherries and cocoa products were not found in literature. Daily intakes of individual lignans were determined by multiplying the daily consumption of individual food items by the respective lignan content in these food items. Individual lignan intakes were summed to obtain total lignan intake.

### 2.5. Statistical Analysis

Descriptive statistics were used for the determination of means, standard deviations (SD), median, percentile and for the percentage analyses of the total and individual lignan dietary intakes.

Odds ratios (ORs) and 95% confidence intervals (CIs) of CVD incidence, hypertension, hypercholesterolemia and obesity according to the lignan intake were computed, using logistic regression analysis, with adjustment for various potential confounders. Where possible, continuous variables were used for the reason of high sensitivity. The regression models included adjustments for age (continuously), education (under-middle, middle, high), BMI (continuous variable), smoking (current, past, never), leisure-time physical activity (low, middle, high), systolic blood pressure (continuously), energy intake (continuously), alcohol intake (continuously), glucose level (continuously), cholesterol level (continuously).

All tests of statistical significance were two-tailed. SAS version 9.2 (SAS Institute, Inc., Cary, NC, USA) was used for all the statistical analyses.

## 3. Results

In the study group of postmenopausal women, the prevalence of CVD was 35.2%, while the prevalence of hypercholesterolemia 82.5%, hypertension 62.9% and central obesity 84.7%.

Table 2, Table 3, Table 4, Table 5 and Table 6 illustrate contributions of food categories and individual food products to the intakes of total and individual lignans. The total lignan intake in the CVD women was 1130.7 μg/day and 1095.1 μg/day in the non-CVD women (*p* = 0.925). Differences in the intake of secoisolariciresinol, pinoresinol, lariciresinol, matairesinol between the groups of women with and without CVD were not statistically significant.

Percentages of individual lignan intakes were also examined. In the CVD women, secoisolariciresinol accounted for 50.15% lignan intake from plant foods, as compared to 44.8% in the control. Pinoresinol, lariciresinol and matairesinol contributed to the total lignan intakes of CVD and non-CVD women in 24.0% vs. 26.1%, 22.7% vs. 26.1% and 3.1% vs. 2.9%, respectively.

Total dietary lignan sources in descending order were: vegetables > nuts and seeds > beverages > fruit > cereals > other food categories > vegetable fats (Table 2). The predominant individual dietary lignan sources for both groups of women included six products: flaxseed, cabbage, tea, potato, legumes, and rye bread for CVD women (~71% of the total intake) and flaxseed, cabbage, tea, potato, legumes, broccoli and cauliflower for non-CVD women (64% of the total lignan intake). The non-CVD women consumed significantly more total lignans from vegetable fats and from foods classified as “other food categories”, although their contribution to total lignan intake was negligible.

The predominant sources of secoisolariciresinol were nuts and seeds in the CVD women (52%) and in the non-CVD women (47%) (Table 3). Dietary SECO sources in descending order were: nuts and seeds > beverages > vegetables > cereals > fruit > other food categories > vegetable fats. Flaxseed and tea were among the individual SECO food sources with the highest impact on its consumption in both groups of women (71% in CVD and 68% in non-CVD). The CVD-free women consumed significantly more SECO from vegetable fats and from “other food categories”, although their contribution to SECO intake was minimum.

The predominant PINO sources both for the CVD and non-CVD women were vegetables (~61% and 56%, respectively) (Table 4). The dietary PINO sources in descending order were: vegetables > fruit > cereals > other food categories > beverages > vegetable fats > nuts and seeds. Of the PINO sources, cabbage intake was the main contributor (43% in CVD and 36% in non-CVD women). The non-CVD women consumed significantly more PINO from vegetable fats and from “other food categories”. Their contribution to PINO intake was minor.

Vegetables were the main source of LARI in both study groups (>65%) (Table 5). Dietary LARI sources in descending order were: vegetables > fruit > cereals > other food categories > nuts and seeds > beverages > vegetable fats. Individual LARI dietary sources were potato and cabbage in both groups of women, and pear in the CVD women (contribution of 50% to total lignans), while broccoli and cauliflower in the controls (contribution 48%). The non-CVD women consumed significantly more LARI from beverages, vegetable fats and from foods classified as “other food categories”, although their contribution to LARI intake was negligible.

Three food groups were substantial MAT sources: vegetables, beverages and cereals in a range of 21.00–32.62% for the individual food group (Table 6). They together accounted for 83.22% of MAT in the CVD and for 79.60% in the non-CVD women. Dietary MAT sources in descending order were: vegetables > beverages > cereals > nuts and seeds > fruit > other food categories > vegetable fats. Tea, legumes and rye bread were the main individual food sources that contributed to MAT intake in 62.7% (CVD) and in 55.03% (non-CVD). The non-CVD women consumed significantly more MAT from vegetable fats and from “other food categories”, although their contribution to MAT intake was negligible.

According to the previous study involving the same participants, women with CVD differed from those without past history of CVD in age, education, smoking habit, cholesterol level, alcohol intake, coffee intake, energy intake, BMI, prevalence of central obesity and hypercholesterolemia ([18] and Table 1). They did not differ in the use of menopause hormone therapy (MHT), leisure-time activity, family history of CVD, consumption of vegetables, fruit and tea, in dietary polyphenol intake (DPI) or dietary antioxidant capacity (DTAC) ([18] and Table 1).

In the multiple adjustment models, total lignans and individual lignans such as pinoresinol, matairesinol and secoisolariciresinol were not associated with the prevalence of CVD, hypercholesterolemia, hypertension and central obesity in the study participants (Table 7).

Of the variables tested in this study, the intake of lariciresinol was associated with the reduced odds of hypercholesterolemia (OR 0.656; 95% CI 0.468–0.921) and hypertension (OR 0.694; 0.505–0.952) (Table 7) in the models adjusted for potential confounders excluding energy intake. When energy intake was added as an adjustment, only OR for lariciresinol and hypercholesterolemia, but not for hypertension, remained statistically significant.

## 4. Discussion

In this group of postmenopausal women, dietary intake of total lignans, as well as the respective lignan types, were not associated with the prevalence of CVD. Recently, we showed an association of the dietary intake of total polyphenols in this group of postmenopausal women with lower prevalence of CVD, but it was not found for the total dietary antioxidant intake [18]. Polyphenols are a large group of phytochemicals that share common structural features of phenolic units. The most prevalent groups of dietary polyphenols are phenolic acids and flavonoids, which are responsible for 97% dietary polyphenol intake, while stilbenes, lignans and other phenolics comprise only the remaining 3% [1]. Thus, the question arises whether and what role lignans can play for human health. Some observational studies suggest that lignans and their derivatives produced by the intestinal bacteria, called enterolignans, provide protection in some types of cancer, diabetes and in cardiovascular disease [23,24,25,26,27,28]. Conversely, this finding was not confirmed by several other studies [10,29,30,31,32]. Lignans may play potentially beneficial roles associated with aging. In middle age, increased lignan intakes were associated with a less cognitive decline [33]. The other roles of dietary lignans remain largely unknown.

The chemical structure of lignans is similar to that of endogenous estrogens. Lignans are considered the major group of phytoestrogens for Western populations [34,35,36], although the intakes of phytoestrogens in these populations are low [36]. Our results 1.131 mg/day and 1.095 mg/day fall within the range of lignan intakes in European females. Total lignan intakes by women in five European countries (Denmark, Finland, Italy, Sweden, United Kingdom) calculated from the intakes of four lignans SECO, LARI, PINO, MAT vary from 1.036 mg/day in Finland to 1.563 mg/day in Sweden [2], with similarity of age range in our study to the age range of 45–79 years in the Swedish Mammography Cohort (SMC) [37]. However, contrary to the SMC, which used food frequency questionnaires as dietary assessment methods, ours and other studies applied dietary records or dietary recalls.

To calculate lignan intakes in this study, we used the Dutch lignan database developed by Milder et al. [20], which was used in several other studies [2,10]. Data gaps were supplemented with lignan values from Kuhnle et al. [21] and Thompson et al. [22]. This approach allowed for the addition of data on lignan contents for most of the food products. Dietary patterns of lignan consumption may differ among countries. We found that the general sources of dietary lignans for Polish postmenopausal women (75–77% intake), considering the amounts that have been ingested, are vegetables (37–38% total dietary intake), nuts and seeds (23–27%) as well as beverages (13–14%), mostly tea. However, in Dutch men and women aged 19–97 years, despite the similarity of primary lignan sources regardless of gender, the lignan sequence was different. Beverages were the first, followed by vegetables, nuts and seeds [38]. In contrast, the main sources of lignans in five European countries according to the Dutch database included cereals and grain products, vegetables, fruit, berries and beverages [2]. In an Italian study which enrolled men and women with a median age of 60 yrs, one third of lignan intake came from wine (mostly red), followed by fruits and vegetables [13].

The main dietary lignan source for postmenopausal women in Poland is flaxseed. This fact can be associated with its laxative properties, and flaxseed is frequently used by the elderly to prevent constipation. Linseeds are also present in bread and breakfast products, as part of the healthy food trend. Flax is the most abundant source of lignans out of other food products. Its content of 335 mg/100 g exceeds almost 200 times that of rye seeds [39].

Among the lignans studied, we found that secoisolariciresinol was the main dietary lignan for postmenopausal Polish women. It contributed 50% to the total lignan intake in the CVD women and 45% in the non-CVD women, as compared to pinoresinol in 24% vs. 26%, lariciresinol 23% vs. 26%, respectively, and matairesinol (3%) in both groups of the women. These findings are in accordance with the individual lignan intakes by middle aged/elderly men and postmenopausal women in Northern Italy, where secoisolariciresinol accounted for 52%, pinoresinol 17%, lariciresinol 27%, and matairesinol 3% of total lignans [13]. However, in Dutch men and women, lariciresinol and pinoresinol contributed to 75% of lignan intake, whereas secoisolariciresinol and matairesinol only for 25% [38]. In our research, the share of pinoresinol and lariciresinol together was no more than 47–52%. The main lignan intake was from secoisolariciresinol, as mentioned above.

The number of studies that have reported on the associations between individual lignan intakes and heart health is limited. Two studies mention matairesinol. The Zutphen Elderly Study, carried out for over 15 years in Dutch elderly men, revealed that the matairesinol intake was inversely associated with the coronary heart disease mortality and cardiovascular disease mortality [10]. On the other hand, in a cross-sectional Italian study, higher matairesinol intake in middle aged/elderly men and postmenopausal women was associated with increased flow mediated dilation [13]. The above studies failed to find significant results for other lignans [10,13]. A recent study showed an inverse relationship between pinoresinol and the prevalence of hypertension in Mediterranean adults [40]. For the first time, our findings suggest that lariciresinol can be protective against hypercholestrolemia in postmenopausal women. Until now, lariciresinol has never been mentioned in the literature in the context of altered lipid levels. However, lariciresinol and matairesinol have been found to be protective against hormone-dependent cancers, such as the ovarian and endometrial ones [23].

Our research shows that the most significant food sources of LARI for Polish postmenopausal women are vegetables (more than 65% in both groups of women), and particularly potato and cabbage. Broccoli and cauliflower are the source of lariciresinol for CVD-free women in contrast to those with CVD. Potatoes are potassium-rich [41], and cruciferous vegetables (Brassica vegetables as cabbages, cauliflower, broccoli) are good sources of vitamin C and phytochemicals (e.g., sulphoraphane), which are essential for CVD prevention [42]. It is likely that some bioactive components present in food, and not only lariciresinol, might account for the cardioprotective effects of these foods. Interestingly, in a prospective study with a mean follow-up of 6.9 years, the consumption of broccoli was associated with insignificant 25–30% reductions in CVD risk in American women [43].

This observational study has strengths and limitations. The strength was the large sample of postmenopausal participants combined from the WOBASZ and WOBASZ II studies. The study groups of CVD and CVD-free women differed by age, education, serum cholesterol levels, smoking habit, BMI and the intakes of alcohol and energy. These differences were taken into account by using the multiple adjustment models. The advantage of this study was the use of several lignan databases, which allowed elimination of most of data gaps.

The main limitation stemmed from the cross-sectional design which does not address the problem of causality. Another limitation was the use of 24-h dietary recalls that may not reflect long-term food consumption, and a validated food frequency questionnaire would be more powerful than a single 24-h dietary recall. Moreover, this study might have underestimated those lignans which were not present in the available lignan databases. Only four lignans were taken into consideration, although other lignans, such as syringaresinol or sesamin can be metabolized to enterolignans. Finally, the overall response rate in the WOBASZ II study was lower than that in the first WOBASZ study (45.5% vs. 76%), which might have had an impact on the study group representativeness.

## 5. Conclusions

This study reinforces the existing concept that the dietary intake of total lignans is not associated with the prevalence of CVD, and provides further evidence that it is not linked to CVD risk factors such as hypertension, hypercholesterolemia and central obesity. However, the intake of the individual lignan, lariciresinol, should be taken into consideration in further studies with regard to its potentially beneficial effect in hypercholesterolemia.

## Figures and Tables

**Table 1:** Descriptive statistics of the studied postmenopausal women with and without CVD *.

	Women Diagnosed with CVD *n* = 916		Women without CVD *n* = 1683		
Characteristics	Mean ± SD	Median (25–75 Percentile)	Mean ± SD	Median (25–75 Percentile)	*p*
Age (years)	65.48 + 9.21	65.00 (58.00–72.00)	60.98 + 8.41	60.00 (55.00–66.00)	<0.0001
Fasting glucose (mmol/L)	5.7 ± 1.9	5.14 (4.72–5.80)	5.5 ± 1.6	5.19 (4.72–5.77)	0.531
Total cholesterol (mmol/L)	5.4 ± 1.2	5.43 (4.64–6.20)	5.8 ± 1.3	5.76 (5.06–6.53)	<0.0001
Energy from food (kcal/day)	1517 ± 580	1438 (1130–1831)	1653 ± 628	1587 (1220–1976)	<0.0001
Intake of vegetables (g/day)	215 ± 156	189 (104–299)	214 ± 155	187 (100–302)	0.850
Intake of fruits (g/day)	214 ± 226	150 (25–300)	217 ± 227	150 (10–320)	0.989
Intake of tea (g/day)	361 ± 252	400 (200–500)	348 ± 251	300 (200–500)	0.180
Intake of coffee (g/day)	130 ± 166	0 (0–250)	175 ± 172	200 (0–250)	<0.0001
Intake of alcohol (pure ethanol g/day)	0.69± 2.67	0.15 (0–0.50)	0.99 ± 2.86	0.25 (0–0.80)	<0.0001
	%		%		
Family history of CVD (%)	39.4		36.1		0.098
Diseases (%)					
Hypertension	73.1		57.3		<0.0001
Myocardial infarction	12.6		0		<0.0001
Stroke	7.9		0		<0.0001
Diabetes	19.8		13.4		<0.0001
Hypercholesterolemia (%)	79.7		84.1		0.0056
BMI [kg/m^2^] (%)					<0.0001
Underweight (BMI < 18.5)	0.3		0.7		
Normal (BMI 18.5–24.99)	20.2		27.8		
Overweight (BMI 25–29.99)	35.2		39.3		
Obesity (BMI > 30)	44.3		32.2		
Central obesity (%)	88.3		82.8		<0.0001
Smoking status (%)					<0.0001
Current smokers	12.5		20.9		
Past smokers	18.6		17.7		
Never smokers	68.9		61.4		
Leisure-time physical activity (%)					0.107
Low level	52.1		47.8		
Middle level	16.5		18.4		
High level	31.3		33.8		
Level of education (%)					0.009
Under middle	61.0		55.2		
Middle	31.9		35.4		
High	7.1		9.5		
Menopause hormone therapy (%)	3.8		4.9		0.178

* The Kruskal–Wallis test was used for continuous variables; the *χ*^2^ test was used for comparisons of categorical variables. BMI: body mass index.

**Table 2:** Contributions of food categories and individual food products to total lignan (LIG) intake in women with and without CVD.

Food Categories		Women with CVD *n* = 916	Women without CVD *n* = 1683	*p* Value
Beverages	μg/day (mean ± SD)	152.5 ± 92	152.9 ± 92	0.7711
	Contribution to LIG (%)	13.49	13.96	
	Major sources (% contribution) *	tea (11.76), coffee (1.17), fruit juices (0.44)	tea (11.72), coffee (1.63), fruit juices (0.44)	
Cereals	μg/day (mean ± SD)	105.8 ± 112	103.9 ± 112	0.5003
	Contribution to LIG (%)	9.36	9.49	
	Major sources (% contribution) *	rye bread (3.82), wheat bread (1.46), mixed bread (1.34)	rye bread (3.66), pastry (3.15), mixed bread (1.77)	
Fruit	μg/day (mean ± SD)	115.9 ± 254	109.6 ± 217	0.5386
	Contribution to LIG (%)	10.25	10.01	
	Major sources (% contribution) *	strawberries (2.17), pears (1.75), peaches (1.54), plums (1.42)	strawberries (2.28), pears (2.22), plums (1.21), peaches (1.00)	
Vegetables	μg/day (mean ± SD)	416.7 ± 524	420.1 ± 538	0.8185
	Contribution to LIG (%)	36.85	38.36	
	Major sources (% contribution) *	cabbage (15.70), potato (7.18), legumes (5.32), carrot (2.13)	cabbage (14.66), potato (8.14), legumes (4.19), broccoli and cauliflower (3.98), carrot (1.90)	
Nuts and seeds	μg/day (mean ± SD)	309.2 ± 4316	252.8 ± 4728	0.5505
	Contribution to LIG (%)	27.35	23.08	
	Major sources (% contribution) *	flaxseed (26.94), sunflower seeds (0.26), walnuts (0.07)	flaxseed (21.63), sesame (0.86), sunflower seeds (0.44)	
Vegetable fats	μg/day (mean ± SD)	14.6 ± 18.4	16.7 ± 19.7	0.0057
	Contribution to LIG (%)	1.29	1.52	
	Major sources (% contribution) *	canola oil (0.66), soft margarine (80% fat) (0.26), sunflower oil (0.22)	canola oil (0.84), soft margarine (80% fat) (0.31), sunflower oil (0.21)	
Other food categories	μg/day (mean ± SD)	16 ± 113	39 ± 460	0.0029
	Contribution to LIG (%)	1.42	3.56	
	Major sources (% contribution) *	confectionery (1.20), cocoa products (0.11)	confectionery (3.31), cocoa products (0.17)	
Total lignan intake	μg/day (mean ± SD)	1130.7 ± 4844	1095.1 ± 4352.7	0.9251
	Contribution to LIG (%)	100	100	
	Major sources (% contribution) *	flaxseed (26.94), cabbage (15.70), tea (11.76), potato (7.18), legumes (5.32), rye bread (3.82)	flaxseed (21.63), cabbage (14.66), tea (11.72), potato (8.14), legumes (4.19), broccoli and cauliflower (3.98)	

* In the total and each food category, only individual food products with the strongest impact on the total lignan intakes were listed.

**Table 3:** Contributions of food categories and individual food products to secoisolariciresinol (SECO) intake in women with and without CVD.

Food Categories		Women with CVD *n* = 916	Women without CVD *n* = 1683	*p* Value
Beverages	μg/day (mean ± SD)	122.3 ± 74.0	122.7 ± 74.0	0.7405
	Contribution to SECO (%)	21.57	25.0	
	Major sources (% contribution) *	tea (19.10), coffee (1.97), fruit juices (0.36)	tea (21.27), coffee (3.10), fruit juices (0.42)	
Cereals	μg/day (mean ± SD)	50.3 ± 68.6	48.8 ± 71.2	0.5669
	Contribution to SECO (%)	8.87	9.94	
	Major sources (% contribution) *	rye bread (5.78), wheat bread (1.21), mixed bread (0.67)	rye bread (6.19), wheat bread (1.43), mixed bread (0.97)	
Fruit	μg/day (mean ± SD)	21.4 ± 42.2	19.7 ± 36.5	0.3650
	Contribution to SECO (%)	3.77	4.01	
	Major sources (% contribution) *	plums (0.94), strawberries (0.46), peaches (0.43)	plums (0.90), strawberries (0.54), apples (0.43)	
Vegetables	μg/day (mean ± SD)	71.3 ± 141.6	62.1 ± 113.8	0.5046
	Contribution to SECO (%)	12.57	12.65	
	Major sources (% contribution) *	legumes (5.41), carrot (2.57), cabbage (1.35)	legumes (4.03), carrot (2.59), cabbage (1.75)	
Nuts and seeds	μg/day (mean ± SD)	296.4 ± 4183	230.9 ± 4567	0.5499
	Contribution to SECO (%)	52.27	47.05	
	Major sources (% contribution) *	flaxseed (52.07), walnuts (0.13), sunflower seeds (0.05)	flaxseed (46.78), walnuts (0.13), sunflower seeds (0.09)	
Vegetable fats	μg/day (mean ± SD)	2.73 ± 4.74	3.19 ± 5.13	0.0113
	Contribution to SECO (%)	0.48	0.65	
	Major sources (% contribution) *	canola oil (0.36), sunflower oil (0.12)	canola oil (0.52), sunflower oil (0.13)	
Other food categories	μg/day (mean ± SD)	5.4 ± 10.8	6.6 ± 11.4	0.0067
	Contribution to SECO (%)	0.95	1.34	
	Major sources (% contribution) *	confectionery (0.39), cocoa products (0.01)	confectionery (0.56), cocoa products (0.02)	
Total secoisolariciresinol	μg/day (mean ± SD)	567.1 ± 4185	490.8 ± 4570	0.7464
	Contribution to SECO (%)	100	100	
	Major sources (% contribution) *	flaxseed (52.07), tea (19.10), rye bread (5.78), legumes (5.41)	flaxseed (46.78), tea (21.27), rye bread (6.19), legumes (4.03)	

* In the total and each food category, only individual food products with the strongest impact on SECO intakes were listed.

**Table 4:** Contributions of food categories and individual food products to pinoresinol (PINO) intake in women with and without CVD.

Food Categories		Women with CVD *n* = 916	Women without CVD *n* = 1683	*p* Value
Beverages	μg/day (mean ± SD)	16.1 ± 11.3	15.7 ± 11.6	0.5580
	Contribution to PINO (%)	5.93	5.49	
	Major sources (% contribution) *	tea (5.30), fruit juices (0.38), beer (0.12), coffee (0.10)	tea (4.86), fruit juices (0.33), beer (0.13), coffee (0.12)	
Cereals	μg/day (mean ± SD)	20.4 ± 20.2	21.7 ± 24.8	0.0901
	Contribution to PINO (%)	7.51	7.59	
	Major sources (% contribution) *	mixed bread (1.96), wheat bread (1.41), breakfast cereals (1.38),	mixed bread (2.32), breakfast cereals (1.58), wheat bread (1.22)	
Fruit	μg/day (mean ± SD)	46.7 ± 129.5	40.7 ± 104.2	0.3223
	Contribution to PINO (%)	17.20	14.23	
	Major sources (% contribution) *	strawberries (4.71), peaches (3.98), plums (3.26)	strawberries (4.55), plums (2.57), peaches (2.40)	
Vegetables	μg/day (mean ± SD)	165.6 ± 312.5	160.7 ± 304.9	0.3921
	Contribution to PINO (%)	60.99	56.19	
	Major sources (% contribution) *	cabbage (43.17), potato (7.40), legumes (4.09)	cabbage (36.19), potato (7.69), legumes (3.95)	
Nuts and seeds	μg/day (mean ± SD)	3.67 ± 47.2	10.2 ± 257.4	0.5429
	Contribution to PINO (%)	1.35	3.57	
	Major sources (% contribution) *	flaxseed (1.22), sunflower seeds (0.11), pumpkin seeds (0.02)	flaxseed (0.90), sesame (2.45) sunflower seeds (0.16)	
Vegetable fats	μg/day (mean ± SD)	11.3 ± 13.4	12.9 ± 14.3	0.0062
	Contribution to PINO (%)	4.16	4.51	
	Major sources (% contribution) *	canola oil (1.89), soft margarine (80% fat) (1.03), sunflower oil (0.62), soft margarine (60% fat) (0.40)	canola oil (2.21), soft margarine (80% fat) (1.13), sunflower oil (0.57), soft margarine (60% fat) (0.33)	
Other food categories	μg/day (mean ± SD)	19.03 ± 83.8	37.0 ± 344.9	0.0249
	Contribution to PINO (%)	7.02	12.93	
	Major sources (% contribution) *	confectionery (2.14), cocoa products (0.24),	confectionery (7.55), cocoa products (0.32),	
Total pinoresinol	μg/day (mean ± SD)	271.5 ± 356.1	286.0 ± 548.0	0.4899
	Contribution to PINO (%)	100	100	
		cabbage (43.17), potato (7.40), tea (5.30)	cabbage (36.19), potato (7.69), confectionery (7.55)	

* In the total and each food category, only individual food products with the strongest impact on PINO intakes were listed.

**Table 5:** Contributions of food categories and individual food products to lariciresinol (LARI) intake in women with and without CVD.

Food Categories		Women with CVD *n* = 916	Women without CVD *n* = 1683	*p* Value
Beverages	μg/day (mean ± SD)	3.45 ± 5.72	3.86 ± 6.14	0.0001
	Contribution to LARI (%)	1.35	1.35	
	Major sources (% contribution) *	fruit juices (0.53), coffee (0.46), tea (0.28)	coffee (0.55), fruit juices (0.44), tea (0.24)	
Cereals	μg/day (mean ± SD)	27.6 ± 44.4	25.9 ± 36.2	0.1919
	Contribution to LARI (%)	10.78	9.06	
	Major sources (% contribution) *	groats and grains (3.85), mixed bread (3.85)	mixed bread (3.45), pastry (3.45)	
Fruit	μg/day (mean ± SD)	45.8 ± 111.4	47.5 ± 106.6	0.5872
	Contribution to LARI (%)	17.88	16.22	
	Major sources (% contribution) *	pear (7.69), strawberries (3.85), citrus fruits (3.85)	pear (6.90), strawberries (3.45), citrus fruits (3.45)	
Vegetables	μg/day (mean ± SD)	168.1 ± 170.6	189.5 ± 241.7	0.1081
	Contribution to LARI (%)	65.64	66.31	
	Major sources (% contribution) *	potato (23.08), cabbage (19.23), broccoli and cauliflower (3.85), tomato (3.85), carrot (3.85)	potato (20.69), cabbage (17.24), broccoli and cauliflower (10.34), tomato (3.45), carrot (3.45)	
Nuts and seeds	μg/day (mean ± SD)	5.39 ± 58.1	7.60 ± 105.7	0.5453
	Contribution to LARI (%)	2.10	2.66	
	Major sources (% contribution) *	flaxseed (1.50), sunflower seeds (0.47), pumpkin seeds (0.1), walnuts (0.02)	flaxseed (1.05), sesame (0.76), sunflower seeds (0.68)	
Vegetable fats	μg/day (mean ± SD)	0.567 ± 0.671	0.644 ± 0.716	0.0062
	Contribution to LARI (%)	0.22	0.23	
	Major sources (% contribution) *	canola oil (0.10), soft margarine (80% fat) (0.05), sunflower oil (0.03)	canola oil (0.11), soft margarine (80% fat) (0.06), sunflower oil (0.03)	
Other food categories	μg/day (mean ± SD)	5.76 ± 26.2	11.44 ± 106.1	0.0263
	Contribution to LARI (%)	2.25	4.00	
	Major sources (% contribution) *	confectionery (1.75), cocoa products (0.22)	confectionery (3.53), cocoa products (0.29)	
Total lariciresinol	μg/day (mean ± SD)	256.1 ± 222.4	285.8 ± 320.5	0.0614
	Contribution to LARI (%)	100	100	
	Major sources (% contribution) *	potato (23.08), cabbage (19.23), pear (7.69)	potato (20.69), cabbage (17.24), broccoli and cauliflower (10.34)	

* In the total and each food category, only individual food products with the strongest impact on LARI intakes were listed.

**Table 6:** Contributions of food categories and individual food products to matairesinol (MAT) intake in women with and without CVD.

Food Categories		Women with CVD *n* = 916	Women without CVD *n* = 1683	*p* Value
Beverages	μg/day (mean ± SD)	10.7 ± 6.8	10.6 ± 6.7	0.8108
	Contribution to MAT (%)	29.72	32.62	
	Major sources (% contribution) *	tea (26.56), fruit juices (1.57), coffee (1.44)	tea (28.37), coffee (2.16), fruit juices (1.70)	
Cereals	μg/day (mean ± SD)	7.56 ± 10.3	7.43 ± 11.1	0.3583
	Contribution to MAT (%)	21.00	22.86	
	Major sources (% contribution) *	rye bread (13.06), wheat bread (2.89), breakfast cereals (2.63)	rye bread (13.42), wheat bread (3.26), breakfast cereals (3.00)	
Fruit	μg/day (mean ± SD)	1.92 ± 7.9	1.69 ± 5.7	0.2993
	Contribution to MAT (%)	5.33	5.20	
	Major sources (% contribution) *	grapes (1.93), citrus fruits (1.44), dried fruits (1.20)	citrus fruits (1.80), grapes (1.74), dried fruits (0.8)	
Vegetables	μg/day (mean ± SD)	11.7 ± 184.6	7.84 ± 99.9	0.7974
	Contribution to MAT (%)	32.50	24.12	
	Major sources (% contribution) *	legumes (23.03), potato (4.28), parsnips (2.72)	legumes (13.60), potato (5.20), parsnips (2.74)	
Nuts and seeds	μg/day (mean ± SD)	3.69 ± 37.2	4.12 ± 45.5	0.5510
	Contribution to MAT (%)	10.25	12.68	
	Major sources (% contribution) *	flaxseed (6.11), sunflower seeds (3.36), pumpkin seeds (0.71), walnuts (0.06)	sunflower seeds (5.97), flaxseed (5.26), pumpkin seed (0.86), sesame (0.52)	
Vegetable fats	μg/day (mean ± SD)	0.0085 ± 0.010	0.0098 ± 0.011	0.0054
	Contribution to MAT (%)	0.02	0.03	
	Major sources (% contribution) *	canola oil (0.01), soft margarine (80% fat) (0.01)	canola oil (0.01), soft margarine (80% fat) (0.01)	
Other food categories	μg/day (mean ± SD)	0.43 ± 2.10	0.82 ± 8.26	0.0033
	Contribution to MAT (%)	1.20	2.52	
	Major sources (% contribution) *	confectionery	confectionery	
Total matairesinol	μg/day (mean ± SD)	36.0 ± 190	32.5 ± 111	0.5174
	Contribution to MAT (%)	100	100	
	Major sources (% contribution) *	tea (26.56), legumes (23.03), rye bread (13.06)	tea (28.37), legumes (13.60), rye bread (13.06)	

* In the total and each food category, only individual food products with the strongest impact on MAT intakes were listed.

**Table 7:** Association between total and individual lignan intake and prevalence of CVD, hypercholesterolemia, hypertension and central obesity; multivariable analysis.

Variables		CVD ^1^ OR (95% CI)	Hypercholeste-Rolemia ^2^ OR (95% CI)	Hypertension ^3^ OR (95% CI)	Central Obesity ^4^ OR (95% CI)
Total lignans (μg/day)	Model 1	1.002 (0.984; 1.020) *p* = 0.8380	0.989 (0.972; 1.006) *p* = 0.2117	0.991 (0.971; 1.011) *p* = 0.3522	0.981 (0.960; 1.003) *p* = 0.0927
	Model 2	1.003 (0.986; 1.021) *p* = 0.7594	0.989 (0.972; 1.007) *p* = 0.2277	0.991 (0.972; 1.011) *p* = 0.3923	0.982 (0.961; 1.003) *p* = 0.0975
Secoisolariciresinol (μg/day)	Model 1	1.003 (0.985; 1.022) 0.7145	0.991 (0.972; 1.009) *p* = 0.3277	0.993 (0.973; 1.013) *p* = 0.4939	0.978 (0.954; 1.002) *p* = 0.0739
	Model 2	1.003 (0.985; 1.022) 0.7136	0.991 (0.972; 1.009) *p* = 0.3229	0.993 (0.973; 1.013) *p* = 0.5003	0.977 (0.953; 1.002) *p* = 0.0742
Pinoresinol (μg/day)	Model 1	0.944 (0.782; 1.139) *p* = 0.5457	0.836 (0.695; 1.005) *p* = 0.0565	0.842 (0.697; 1.016) *p* = 0.0734	1.126 (0.881; 1.455) *p* = 0.3665
	Model 2	0.987 (0.821; 1.187) *p* = 0.8927	0.853 (0.708; 1.028) *p* = 0.0958	0.868 (0.720; 1.047) *p* = 0.1382	1.160 (0.886; 1.520) *p* = 0.2807
Lariciresinol (μg/day)	Model 1	0.717 (0.507; 1.014) *p* = 0.0597	0.656 (0.468; 0.921) *p* = 0.0148	0.694 (0.505; 0.952) *p* = 0.0236	1.048 (0.713; 1.539) *p* = 0.8128
	Model 2	0.815 (0.575; 1.154) *p* = 0.2487	0.693 (0.488; 0.985) *p* = 0.0410	0.744 (0.539; 1.029) *p* = 0.0736	1.109 (0.741; 1.659) *p* = 0.6146
Matairesinol (μg/day)	Model 1	1.347 (0.776; 2.338) *p* = 0.2902	1.082 (0.459; 2.552) *p* = 0.8576	1.290 (0.692; 2.405) *p* = 0.4228	1.128 (0.451; 2.817) *p* = 0.7968
	Model 2	1.415 (0.811; 2.469) *p* = 0.2214	1.133 (0.463; 2.777) *p* = 0.7842	1.347 (0.708; 2.564) *p* = 0.3645	1.166 (0.455; 2.989) *p* = 0.7489

OR: odds ratio; ^1^ CVD: Model 1—adjusted for age, smoking, BMI, alcohol intake, education, leisure-time physical activity, SBP, glucose level, cholesterol level, menopause hormone therapy. Model 2—additionally adjusted for energy intake. ^2^ Hypercholesterolemia: Model 1—adjusted for age, smoking, BMI, alcohol intake, education, leisure-time physical activity, SBP, glucose level, menopause hormone therapy. Model 2—additionally adjusted for energy intake. ^3^ Hypertension: Model 1—adjusted for age, smoking, BMI, alcohol intake, education, leisure-time physical activity, glucose level, cholesterol level, menopause hormone therapy. Model 2—additionally adjusted for energy intake. ^4^ Central obesity: Model 1—adjusted for age, smoking, alcohol intake, education, leisure-time physical activity, SBP, glucose level, cholesterol level, menopause hormone therapy. Model 2—additionally adjusted for energy intake.
